# The Impact of Prior Information on Estimates of Disease Transmissibility Using Bayesian Tools

**DOI:** 10.1371/journal.pone.0118762

**Published:** 2015-03-20

**Authors:** Carlee B. Moser, Mayetri Gupta, Brett N. Archer, Laura F. White

**Affiliations:** 1 Department of Biostatistics, Boston University School of Public Health, Boston University, Boston, Massachusetts, United States of America; 2 National Institute for Communicable Diseases, National Health Laboratory Service, Johannesburg, South Africa; National Institute for Public Health and the Environment, NETHERLANDS

## Abstract

The basic reproductive number (R₀) and the distribution of the serial interval (SI) are often used to quantify transmission during an infectious disease outbreak. In this paper, we present estimates of R₀ and SI from the 2003 SARS outbreak in Hong Kong and Singapore, and the 2009 pandemic influenza A(H1N1) outbreak in South Africa using methods that expand upon an existing Bayesian framework. This expanded framework allows for the incorporation of additional information, such as contact tracing or household data, through prior distributions. The results for the R₀ and the SI from the influenza outbreak in South Africa were similar regardless of the prior information (${\hat{R}}_{0}$ = 1.36–1.46, $\hat{\mu }$ = 2.0–2.7, $\hat{\mu }$ = mean of the SI). The estimates of R₀ and μ for the SARS outbreak ranged from 2.0–4.4 and 7.4–11.3, respectively, and were shown to vary depending on the use of contact tracing data. The impact of the contact tracing data was likely due to the small number of SARS cases relative to the size of the contact tracing sample.

## Introduction

When an infectious disease outbreak occurs, public health officials need to understand the dynamics of disease transmission in order to launch an effective response. Two quantities that are often used to describe transmission are the basic reproductive number and the distribution of the serial interval (SI). The basic reproductive number (R_0_) is the average number of secondary cases a primary case will infect, assuming a completely susceptible population [1]. The reproductive number is always nonnegative; values less than one are indicative of an outbreak that will not continue to grow in the absence of imported cases. When R_0_ is larger than one, the magnitude of the value guides the types of control measures that are necessary to restrict transmission and control the outbreak. It is also essential to understand the timing between primary and secondary cases. For a given R_0_, if secondary cases occur shortly after the primary cases, a rapidly growing outbreak will result, which can be more difficult to control than an outbreak with a longer time interval between cases. The timing of the secondary cases is most easily measured by the SI distribution, an observable quantity. The SI is defined as the time between symptom onset in successive cases in a chain of backward transmission. The SI is used as a surrogate measure for the generation interval, which is unobservable, and is defined as the time between consecutive infections in the chain of transmission [2]. R_0_ and the SI distribution provide important information that is used to initiate an appropriate public health response to an infectious disease outbreak.

Many methods exist to quantify the R_0_ and the SI [3]. Typically, the SI distribution is estimated using contact tracing or household data (see for example [4–5]); however, these studies are often small and subject to potential bias and errors in recall by participants. White and Pagano [6] introduced a novel approach to simultaneously estimate the R_0_ and the SI using only data from the epidemic curve. In recent years Bayesian methods have been developed to estimate transmission parameters, and can be particularly useful in instances with sparse data or when prior data about an outbreak exists; however these types of models have often been limited to Bayesian evidence synthesis or compartmental models [7–12]. Becker et al. [13] introduced a Bayesian framework to estimate R_0_ and the SI distribution by augmenting the likelihood function, introduced by White and Pagano, with independent observations of the SI from contact tracing data, and obtained posterior estimates through MCMC methods. They also made recommendations about the number of observations from the epidemic curve and contact tracing sample needed to obtain reliable estimates for R_0_ and the SI distribution.

In this paper we describe an extension of the Bayesian methods introduced by Becker et al. Our approach, like Becker et al., also allows for the inclusion of additional data, but does so through a different mechanism, as prior information via prior distributions. In what follows, we present the statistical model introduced by White and Pagano, and outline how to include additional information, such as contact tracing data, via the prior distributions. Details of a simulation study that examines our method are also discussed. Finally, we analyze data from the 2003 SARS outbreak in Hong Kong and Singapore, and the 2009 pandemic influenza A(H1N1) outbreak in South Africa with our method.

## Methods

### Statistical model

The method proposed in White and Pagano [6] can simultaneously estimate the R_0_ and the SI by maximizing the likelihood shown in equation 1.

$$L\left(R_{0},p\right)=\prod_{t=1}^{T}\frac{e^{-\mu_{t}}\mu_{t}^{N_{t}}}{N_{t}}\;,\;\mu_{t}=R_{0}\sum_{j=1}^{\text{min}\;\left(k,t\right)}N_{t-j}p_{j}$$ (1)

The number of new secondary cases at a given time *t* is defined as N_t_. For simplicity, we assume *t* indexes days. Here R_0_ is the basic reproductive number and p_i_ describes the probability of a serial interval that is *i* days long. The serial intervals are constrained to be no longer than *k* day and, to follow a multinomial distribution, which is assumed to be stationary. We perform estimation using a Markov chain Monte Carlo (MCMC) method using OpenBUGS software via the BRugs package in R version 2.11.1 [14–16]. See S4 Appendix for details.

### Prior Distributions

To model an outbreak using a Bayesian method, prior distributions are necessary for each parameter of interest: in this instance R_0_ and **p** = {p_i_,…, p_k_}. We choose the prior distribution for R_0_ to be log-normal, such that log(R_0_) is distributed Gaussian with mean 0 and variance 1,000.

Because we model a discrete form of the serial interval distribution, like White and Pagano and Becker et al., a natural choice for a prior distribution for **p** is the Dirichlet distribution, which is the conjugate prior for the multinomial distribution. The Dirichlet distribution,

$$f\left(p|\alpha \right)=\frac{\Gamma (\sum_{i}\alpha_{i})}{\prod_{i}\Gamma (\alpha_{i})}p_{1}^{\alpha_{1}-1}p_{2}^{\alpha_{2}-1}\ldots p_{k}^{\alpha_{k}-1},$$ (2)

is parameterized by a set **α**, known as hyperparameters, with each α_i_ corresponding to a specific p_i_. In the absence of prior information, we want to select hyperparameters for the Dirichlet prior such that the distribution will be noninformative [17–22]. We examined different priors such as Jeffrey’s prior, which assigns 0.5 for all values of α_i_, and the Bayes-Laplace prior, which assigns 1 to all α_i_ values. We found that using hyperparameters that are less than 1 can result in a lack of convergence or poor mixing. Because of this, when no additional information about the SI distribution is available, we select the Bayes-Laplace prior. This choice is not noninformative, as it imposes a uniform distribution on the prior, but it is reasonable for outbreaks where there are no contact tracing samples. This prior will be referred to as the uniform prior in the following sections.

When additional information is available, such as contact tracing from initial cases, we can inform the prior distribution with these observations. The hyperparameters of the Dirichlet distribution can be updated with the number of observed serial intervals corresponding to each day of the SI. Previously, Becker et al. [13] incorporated this information by augmenting it with the likelihood (in equation 1) and used a Bayes-Laplace prior distribution for the SI (see equation 3). Our approach directly informs the SI prior distribution with the observed SIs, which enables us to guide the estimation of the SI by weighting each day of the SI according to the observed serial intervals. The Bayesian model considers the prior information in the estimation, but still puts emphasis on the epidemic curve (daily incidence data).

To further explain the differences between the approaches the following simplified model formulation can be used. The posterior distribution presented in Becker et al. can be generalized to: *P*(*R*_0_,*p*|*N*,*r*) ∝ *P*(*R*_0_) * *P*(*p*) * *L*_1_(*N*|*R*_0_,*p*)* *L*_2_(*r*|*p*), where the posterior is proportional to the prior distributions and likelihood function. P(R_0_) and P(p) represent the prior distributions for R_0_ and p, and L_1_ and L_2_ are the components of the likelihood, where L_1_ is from White and Pagano and L_2_ is the contact tracing sample that is used to augment the White and Pagano method. In this formulations, P(p) ∼ Dirichlet(**α = 1**).

The posterior distribution that we propose has a slightly different formulation, in that the contact tracing data is incorporated in the SI prior: *P*(*R*_0_,*p*|*N*,*r*) ∝ *P*(*R*_0_) * *P*(*p*|*r*) * *L*_1_(*N*|*R*_0_,*p*). In our approach, the likelihood function only considers data from the epidemic curve, N, and the contact tracing sample is used to inform the hyperparameters of the SI prior, P(p|r) ∼ Dirichlet(**α = r**). With this formulation we can consider instances when contact tracing is not available, P(p|r) ∼ Dirichlet(**α = 1**), or we can weight the contact tracing sample as appropriate, P(p|r) ∼ Dirichlet(**α = r**_**w**_).

Our method is flexible because it does not require independent observations of the SI from the same population. For example, when a contact tracing sample is not available, a sample from another outbreak of the same or similar pathogen could be used to inform the prior. If the contact tracing is obtained from a population that is different from the current outbreak population or the contact tracing sample is very large relative to the current outbreak, the contact tracing could be down weighted in the prior, thus putting more emphasis on the current outbreak data as opposed to the outside source. It may not be appropriate to combine data sources from different populations via the likelihood function, as in Becker et al., and instances with large contact tracing samples relative to the outbreak size could bias the results.

If contact tracing samples are not available for an analysis, then the uniform prior (Dirichlet(**α = 1**)) can be used instead; however, deciding the maximum length of the SI, k, may not be obvious. The deviance information criteria (DIC), a model assessment tool, can be used in these situations to select the best model. A range of plausible values for k should be selected and a model based on each k should be analyzed. The fit of each model can then be compared with the DIC; smaller DIC indicates better model fit. The DIC is useful to compare different models because it uses the posterior densities in its calculation, accounts for the complexity of the model and can easily be implemented in an MCMC simulation [23].

### Simulation Study

The following simulation study was designed to assess the validity and utility of our proposed approach and provide guidance in selecting prior distributions. A description of the simulations, including how the data are generated and summarized is included next.

#### Description of Simulations

**Fig 1:**
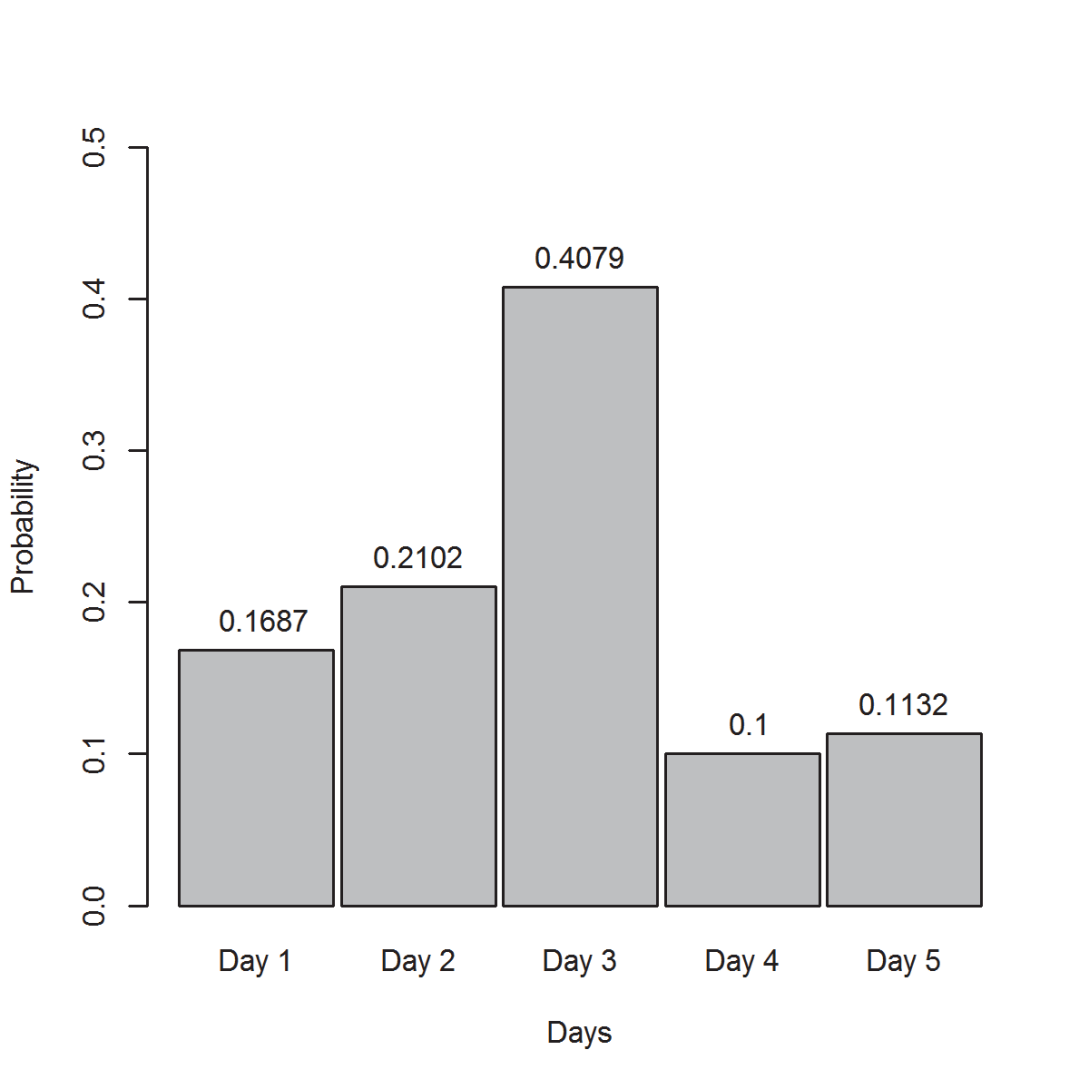
Serial Interval Distribution for Simulated Outbreaks.

We generate outbreaks in the same manner as suggested by White and Pagano [6], and model our simulated epidemics to be similar to influenza by setting the SI to have a maximum length 5 and mean of 2.74 (see Fig. 1), with parameters selected based on estimates from CDC influenza data [24]. R_0_ assumes the values of 1.25, 3, and 6, and we examine epidemic sizes of 50, 200 and 500 cases. For each scenario discussed, 300 epidemics are generated, with all cases on the first day assumed to be index cases. A description of the epidemic curves for each scenario is shown in Table 1.

**Table 1:** Means and Ranges of the Epidemic Lengths for each Simulation Scenario.

	R_0_ = 1.25		R_0_ = 3		R_0_ = 6	
N = 500	50	25–123	13	11–19	8	7–11
N = 200	40	21–96	11	8–21	7	6–9
N = 50	24	9–59	8	6–15	5	4–7

There are 300 Epidemics Generated for each Scenario.

The data from these nine scenarios are analyzed using the White and Pagano model (equation 1) with k = 5 and the prior distributions outlined in Table 2. We first include the uniform prior previously discussed (prior 1), which represents the situation when contact tracing data is not available and we only have daily incidence data (epidemic curve). Two informative prior distributions are also included, where the hyperparameters of the prior (observed SIs) match the SI distribution used to generate the data (Fig. 1). The first informative prior (prior 2) has 20 observed SIs, which corresponds to 40%, 10%, and 4% of the epidemic sizes listed above; the second (prior 3) includes only 10 observed SIs, which corresponds to 20%, 5%, 2% of the epidemic sizes. These priors (priors 2 and 3) are considered the “gold standard” in our simulation because the distribution of SI observations matches the SI distribution used to generate the data. We also consider the situation when the contact tracing data used to inform the prior is biased, which is possible in a real life situation. The first misspecified prior (prior 4) is informed using an SI distribution that differs from the true distribution; however, the overall shape and mean of the SI distribution (μ = 2.8) are very similar to the distribution used to generate the data. This scenario could occur when contact tracing data is included from another study of the same or similar pathogen. The second misspecified prior (prior 5) has a SI distribution that differs dramatically from the distribution used to generate the data, with a heavy tail and a prior mean of four (μ = 4). Both priors 4 and 5 are composed of 20 observed SIs, which corresponds to 40%, 10% and 4% of the epidemic sizes.

**Table 2:** Dirichlet Prior Distributions for the Serial Interval for the Simulation Study.

Prior	Type	Hyperparameters
1	Uniform	**α** = (1, 1, 1, 1, 1)
2	*Ideally* Specified	**α** = (3, 4, 10, 1, 2)
3	*Ideally* Specified	**α** = (1, 2, 5, 1, 1)
4	Misspecified	**α** = (1, 10, 4, 2, 3)
5	Misspecified	**α** = (1, 2, 3, 4, 10)

A second set of analyses examines in more detail the scenario when contact tracing data is not available and the investigator must choose the maximum length of serial interval. These analyses model the simulated data assuming various lengths of k, k = {5, 7, 10, 15, 20} and focus on outbreaks based on R_0_ = 1.25 and N = 500 and 200. We limit these analyses to only these outbreaks because of outbreak length limitations, that is, the maximum k considered (k = 20) is larger than some simulated outbreak lengths, which limits the number of observable generations of transmission (see Table 1). The uniform prior with corresponding k is utilized for each model.

Last, we analyze the simulated data using the Becker et al. method [13]. The Becker likelihood function, shown in equation 3, is based on the White and Pagano model (equation 1) and is augmented by an additional component composed of observed SIs from contact tracing data. The r_j_ r_j_ represents the number of observed serial intervals for each increment of the serial interval distribution.

$$L\left(R_{0},p\right)=\prod_{j=1}^{k}p_{j}^{r_{j}}\prod_{t=1}^{T}\frac{e^{-\mu_{t}}\mu_{t}^{N_{t}}}{N_{t}!}\;,\;\mu_{t}=R_{0}\sum_{j=1}^{\text{min}\;\left(k,t\right)}N_{t-j}p_{j}$$ (3)

For the analyses with the Becker method we also select prior distributions for the parameters, and as with our approach the prior distribution for the reproductive number is log-normal. The serial interval prior for the Becker method differs from our approach because it remains the same across all scenarios, and is set to be a Dirichlet distribution with **α** = (1, 1, 1, 1, 1), as shown in their paper. The contact tracing data is incorporated in the likelihood function, and thus Table 2 contains the information used to augment the Becker likelihood function (equation 3). We assume that these data are independent observations of the SI for the simulation analysis.

We analyze all the simulations via MCMC methods and provide posterior summaries. For each simulated outbreak, single chains were run for 20,000 iterations with a 10,000 iteration burn-in. Trace plots, density plots, and autocorrelation plots were examined to explore sampler convergence.

### Description of Outbreak Data

We apply our method to data from the 2009 pandemic influenza A(H1N1) outbreak in South Africa and to the 2003 SARS outbreak in Hong Kong and Singapore.

#### South Africa Influenza A(H1N1)2009pdm

**Fig 2:**
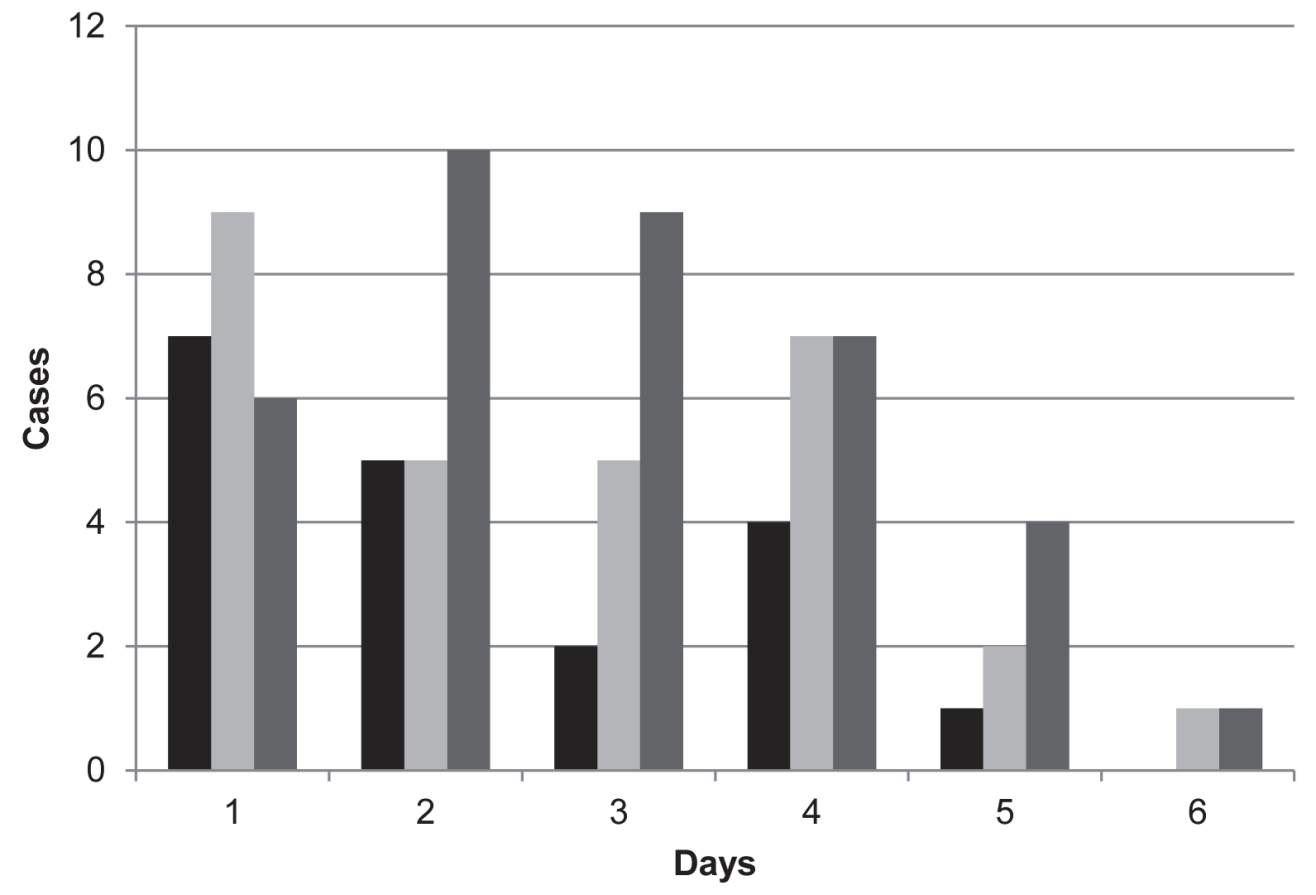
Contact tracing data from influenza A(H1N1)2009pdm. Black: Confirmed ILL; Light Gray: Probable ILL; Dark Gray: Australia A(H1N1) Data.

The National Institute for Communicable Diseases (NICD) of the National Health Laboratory (NHLS) in South Africa maintained a database of laboratory confirmed cases of H1N1 during the 2009 pandemic. Data were collected throughout the country beginning in April and lasting until October, and include basic demographic information, and spatial and temporal data for each case; date of symptom onset was imputed using a multiple imputation approach for some cases [25–27]. We randomly select one imputed outbreak, and because we are interested in estimating R_0_, only the initial epidemic growth phase of the outbreak is examined. This includes 2,423 cases from days 15 to 35 of the epidemic. Large gaps in cases occurred during the first two weeks of the epidemic due to no sustained transmission from these presumably imported cases. Therefore, only cases occurring after sustained transmission was established are included. Three possible contact tracing samples are considered for informing the SI prior distribution, as shown in Fig. 2. The first two samples are from contact tracing data collected in South Africa during the pandemic and consist of confirmed and probable influenza-like illness (ILI) secondary cases [26]. The third set of data is empirically observed SIs from the influenza A(H1N1)2009pdm outbreak in Victoria, Australia [28].

#### Severe Acute Respiratory Syndrome (SARS)

Severe acute respiratory syndrome (SARS) first appeared in Hong Kong and Singapore in 2003. Reported cases of SARS and the date of onset were collected and reported by the World Health Organization (WHO) for Hong Kong and Singapore [29]. We focus our analysis on the initial growth phase in order to estimate R_0_. Hong Kong experienced 659 cases in the first 39 days of the outbreak (February 15^th^—March 25^th^), while Singapore had 57 cases in the first 20 days of the outbreak (February 25^th^—March 16^th^) [29]. The SI prior distribution is based on 179 (314% of the Singapore incidence data and 27% of the Hong Kong data) observed SIs in Singapore with a mean SI of 8.41 days [30].

## Results

### Simulation Results

Detailed results from the simulations are provided in the supplement and are displayed in S1 and S2 Fig. and S1-S12 Tables in S2 Appendix, and in S1 Appendix.

In general, our simulations show that our approach provides good estimation of ${\hat{R}}_{0}$ and $\hat{\mu }$ when we include contact tracing data that is in agreement with the outbreak data, regardless of the outbreak size. However, the estimates for the reproductive number were affected by the number of observed generations of transmission. When outbreak sizes were small and reproductive numbers were large we observed short epidemic lengths, such as when N = 50 and R_0_ = 6 we only observe epidemic lengths of four to five days for an SI with maximum length five. We see for some scenarios with R_0_ = 6, that ${\hat{R}}_{0}$ is slightly underestimated, which is likely due to the limited number of observed generations of transmission and limited total outbreak lengths (see Table 2).

There were inconsistent results when using a misspecified prior based on biased contact tracing data. When informing the prior with a contact tracing sample that had a total number of observed SIs equaling more than 4% of the total outbreak size, the estimates were biased (i.e. simulations with N = 50 and N = 200). Because we will never know if the contact tracing data is in agreement with the epidemic curve, it is important to not weight the SI prior to be more than 4% of the total outbreak size. In general, if enough generations of transmission are observed, the prior weight has less impact; however, if the total epidemic length is relatively short and few generations of transmission have been observed, then a smaller weight of 1–2% may be more appropriate.

**Fig 3:**
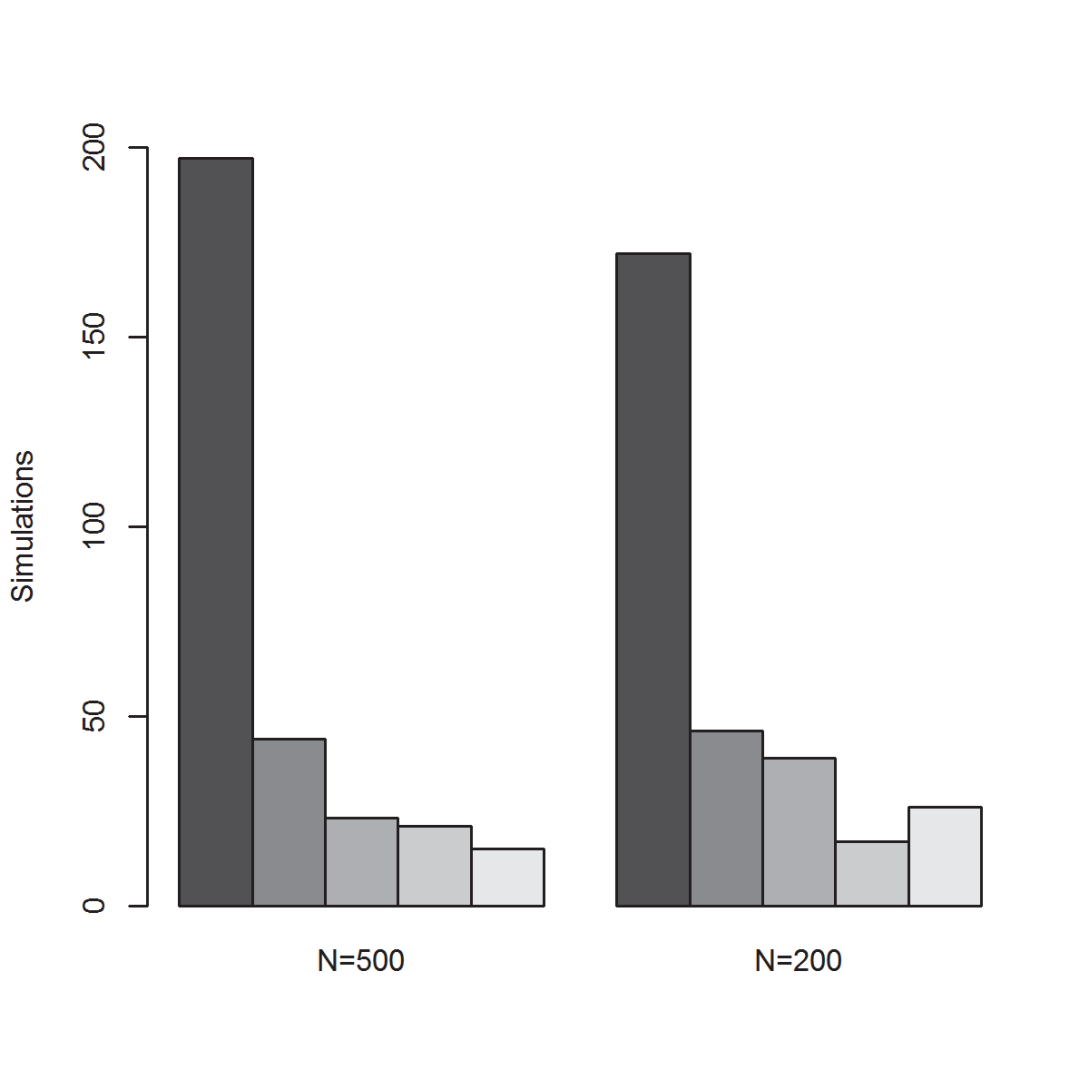
Summary of DIC-based Prior Selection for Simulated Outbreaks. Gray scale from dark to light: k = 5, k = 7, k = 10, k = 15, k = 20.

The comparison of the models with varying values of k, using uniform SI priors, shows that the DIC selects the model with k = 5 most often. To summarize the simulations with the DIC, the minimum DIC was calculated across models and then the frequency that each model was selected is depicted in Fig. 3. Models with larger ks were sometimes selected; however, the differences between the DICs were small in these instances. The ranges of epidemic curve lengths for the two scenarios that were considered are 25–123 days (N = 500) and 21–96 days (N = 200). Some outbreak lengths are small relative to the largest maximum SI length (k = 20) that was considered, which could affect the estimates. However, we did observe that the DIC is a useful model selection tool.

#### Simulation Conclusions

When contact tracing samples are available this data should be used to inform the SI prior distribution. Careful thought should be given to the size of the prior relative to the total outbreak size and length. For smaller outbreaks it is especially important to make sure that the contact tracing sample size is not too large, which could potentially bias the estimates. If enough generations of transmission have been observed to properly estimate a SI of a given maximum length k, then the SI prior should be weighted to be no more 4% of the total outbreak size; however, a range of weights could be considered to examine the robustness of the results. When total outbreak lengths are limited, weighting the SI prior to be 1–2% of the total outbreak size may be more appropriate. If contact tracing data is not available then a range of possible models with reasonable choices of k should be considered. The choice of k can be determined by prior knowledge of similar outbreaks or general knowledge about the pathogen. Each model can be analyzed using a uniform prior distribution for the SI and the models can be compared using the DIC (or other model assessment tool). Smallest DICs indicate best fit, which can be used to narrow down the best model and value for k. These estimates can also be updated and confirmed as the outbreak continues and more cases are observed.

### Outbreak Data

#### South Africa Influenza A(H1N1)2009pdm

The results of the South African influenza A(H1N1)2009pdm outbreak are shown in Table 3. Five separate MCMC analyses were conducted, each with a different SI prior distribution, as well as two additional analyses using the White and Pagano method. The first three MCMC analyses were performed using the informative priors shown in Fig. 2, and are composed of the actual number of observed serial intervals from the contact tracing samples, which corresponds to 0.8–1.5% of the total outbreak size. The confirmed influenza-like illness cases have a maximum SI length of five days, while the probable cases have a maximum length of six days. Two uniform prior distributions were included, with five and six day maximum SI lengths, chosen to match the lengths of contact tracing samples. To assess if these models (choices of k) are appropriate, additional models with varying values of k, using uniform priors, were also included. Values of k that were considered ranged from k = 5 to 20, with model comparison done via the DIC (Table 4).

**Table 3:** Means and 95% CIs for South Africa Influenza A(H1N1)2009pdm.

	Informative Prior			Uniform Prior		White and Pagano	
	C ILI	P ILI	Australia	K = 5	K = 6	K = 5	K = 6
R_0_	1.36	1.41	1.43	1.38	1.46	1.36	1.47
	1.27, 1.45	1.32, 1.51	1.35, 1.53	1.28, 1.49	1.34, 1.60	1.06, 2.65	1.08, 2.81
μ	2.00	2.33	2.42	2.19	2.66	2.07	2.70
	1.62, 2.42	1.92, 2.81	2.06, 2.85	1.66, 2.67	2.03, 3.29	1.08, 3.70	1.35, 4.37
p_1_	0.55	0.45	0.42	0.56	0.49	0.57	0.50
	0.43, 0.67	0.38, 0.61	0.31, 0.52	0.43, 0.70	0.35, 0.63	0.08, 0.96	0.10, 0.85
p_2_	0.19	0.15	0.21	0.15	0.15	0.22	0.20
	0.09, 0.32	0.07, 0.25	0.12, 0.31	0.04, 0.29	0.04, 0.28	0.00, 0.54	0.00, 0.49
p_3_	0.04	0.08	0.11	0.03	0.03	0.00	0.00
	0.01, 0.11	0.03, 0.15	0.06, 0.19	0.00, 0.10	0.00, 0.09	0.00, 0.42	0.00, 0.38
p_4_	0.12	0.14	0.11	0.05	0.04	0.00	0.00
	0.04, 0.23	0.06, 0.24	0.05, 0.20	0.00, 0.18	0.00, 0.13	0.00, 0.41	0.00, 0.32
p_5_	0.09	0.07	0.11	0.20	0.10	0.21	0.00
	0.00, 0.22	0.01, 0.18	0.04, 0.20	0.04, 0.35	0.01, 0.26	0.00, 0.57	0.00, 0.39
p_6_		0.06	0.04		0.18		0.30
		0.00, 0.18	0.00, 0.14		0.02, 0.35		0.00, 0.61

Australia: Australian Contact Trace data; C ILI: Confirmed Influenza-Like Illness; P ILI: Probable Influenza-Like Illness. Informative and Uniform priors are 95% credible intervals, White and Pagano Results are 95% Confidence Intervals.

**Table 4:** DIC, Means and 95% CIs for South Africa Influenza A(H1N1)2009pdm for various uniform priors.

	DIC	R_0_	μ
K = 5	321.4	1.38	2.19
		1.28, 1.49	1.66, 2.67
**K = 6**	**315.9**	**1.46**	**2.66**
		**1.34, 1.60**	**2.03, 3.29**
**K = 7**	**316**	**1.51**	**2.92**
		**1.37, 1.66**	**2.23, 3.61**
K = 8	317.3	1.53	3.04
		1.38, 1.69	2.33, 3.75
K = 9	318.7	1.56	3.21
		1.41, 1.72	2.50, 3.92
K = 10	319.7	1.57	3.33
		1.43, 1.75	2.60, 4.09
K = 11	320.6	1.60	3.53
		1.44, 1.78	2.75, 4.34
K = 12	321.5	1.63	3.75
		1.46, 1.83	2.89, 4.70
K = 13	321.3	1.69	4.19
		1.50, 1.92	3.19, 5.37
K = 14	320.7	1.77	4.76
		1.55, 2.04	3.54, 6.17
K = 15	321	1.82	5.11
		1.59, 2.10	3.82, 6.54
K = 16	321.6	1.87	5.44
		1.61, 2.17	4.06, 6.95
K = 17	321.2	1.94	5.98
		1.67, 2.27	4.49, 7.62
K = 18	321.4	2.01	6.45
		1.71, 2.36	4.82, 8.20
K = 19	320.9	2.10	7.06
		1.77, 2.50	5.27, 9.00
K = 20	320.3	2.19	7.71
		1.82, 2.67	5.71, 9.92

R_0_ was estimated between 1.36–1.46 for when using the priors based on observed SIs from contact tracing, which is consistent with other estimates from South Africa [27, 31]. The estimates for the average serial interval range from 2.00–2.66 days. Slightly larger posterior estimates for μ and R_0_ are observed for the analyses with a six-day maximum SI length, but overall the results are similar between approaches. The results from the White and Pagano maximum likelihood estimation (MLE) method are consistent with those from the Bayesian models. The MLEs for the five-day SI and six-day SI are ${\hat{R}}_{0}$ = 1.36 and 1.47, and $\hat{\mu }$ = 2.07 and 2.70, respectively. The confidence intervals for White and Pagano are wider than the credible intervals obtained from the Bayesian approach, but otherwise they are similar.

The DIC assessment compared 16 possible models with differing values of k, each with a uniform prior. We observe the smallest DIC for models with k = 6 or k = 7, which supports our findings above and our choice of k. Additional exploratory analyses are included in S3 Appendix, which examine the results when using a weighted contact tracing sample.

#### Severe Acute Respiratory Syndrome (SARS)

Singapore and Hong Kong SARS data are analyzed separately for each location and the results are shown in Table 5. For each location, two analyses are conducted: the first using a contact tracing informed prior distribution for the SI, and the second using a uniform prior distribution, both with a maximum length of 20 days. Additionally, both outbreaks are analyzed with the White and Pagano method. The R_0_ and μ posterior estimates vary depending upon if contact tracing is used as part of the prior. In both locations, there are larger estimates for R_0_ and μ from the analyses using the uniform prior compared to the informed prior (Singapore: ${\hat{R}}_{0}$ = 4.43 v. 3.86, $\hat{\mu }$ = 10.68 v. 8.45; Hong Kong: ${\hat{R}}_{0}$ = 2.78 v. 2.02, $\hat{\mu }$ = 11.33 v. 7.42). The results for the informative Singapore analysis are similar to those observed in Lipsitch et al. [30], who observed a mean SI of 8.4 days. The epidemic curve data from Singapore is primarily composed of cases prior to March 12^th^, the date in which WHO released their first global SARS alert. Lipsitch et al. noted that the mean SI was slightly larger before this date, with μ = 10 days, which is consistent with our results for the uniform prior. Our estimate of R_0_, 3.86 (95% CI: 2.86–5.01), is similar to the estimate calculated by Wallinga and Teunis before the global health alert, ${\hat{R}}_{t}$ = 3.1 (95% CI: 2.3, 4.0), and to the R_0_ calculated by Lipsitch et al., ${\hat{R}}_{0}$ = 3.5 (95% CI: 1.5, 7.7). The Hong Kong results are slightly different than those from Singapore, but a similar trend is seen when including contact tracing data. Our estimates for R_0_ (2.02 for the informative prior; 2.78 for the uniform prior) are smaller than what Wallinga and Teunis calculated (${\hat{R}}_{t}$ = 3.6, 95% CI: 3.1, 4.2). The estimates from White and Pagano are substantially larger, especially for Singapore, which is likely to be due to the small sample size and large number of parameters. The 95% confidence intervals estimates are also wide.

**Table 5:** Means and 95% CIs for SARS outbreak.

	Singapore		Hong Kong	
	R_0_	μ	R_0_	μ
Informative Prior	3.86	8.45	2.02	7.42
	2.56, 5.01	7.91, 9.01	1.83, 2.22	6.77, 8.11
Uniform	4.43	10.68	2.78	11.33
	2.63, 7.19	8.23, 13.24	2.19, 3.55	9.27, 13.39
White and Pagano	23.95	16.86	4.93	15.73
	16.67, 49.27	15.10, 18.39	3.54, 26.71	13.78, 18.55

Informative and Uniform priors are 95% credible intervals, White and Pagano Results are 95% Confidence Intervals.

The results from the analysis of the Singapore data should be interpreted with caution given the limited outbreak size and short total epidemic length. The Hong Kong outbreak was larger than Singapore, but was still limited in terms of total length and was not substantially larger than the contact tracing sample size; the contact tracing sample size was 27% of the total outbreak size. We would recommend down weighting the contact tracing sample in the prior in this instance, but due to the large k this may not be feasible. The simplest prior we consider is the uniform prior and with k = 20 this is already equivalent to 3% of the total outbreak size, implying that any informative prior would be weighted to be at least 3% of the total outbreak size. Because of this, the uniform prior may be most appropriate for this analysis.

The SARS outbreak in Hong Kong was analyzed using three models with varying lengths of k (k = 10, 15, and 20) and a uniform prior for the SI; Singapore was not included due to the short total outbreak length and larger values for k were not considered due to a limited epidemic curve length of 39 days in Hong Kong. The models were compared via the DIC and showed that the model with k = 20 was a better fit than k = 10 or k = 15. For k = 10, the DIC = 323.9, with ${\hat{R}}_{0}$ = 1.45 and $\hat{\mu }$ = 3.40; for k = 15, the DIC = 289.6, with ${\hat{R}}_{0}$ = 2.02 and $\hat{\mu }$ = 8.12; for k = 20, the DIC = 282.2, with ${\hat{R}}_{0}$ = 2.78 and $\hat{\mu }$ = 11.33. Modeling the SI with maximum length 20, we see that estimates for R_0_ = 2–3 and the mean SI = 8–11 days.

## Discussion

In this paper, we modified the Bayesian framework introduced by Becker et al. to obtain robust estimates of the reproductive number and the SI distribution using the White and Pagano [6] likelihood function. This framework allows for the inclusion of additional data sources beyond the epidemic curve through the prior distributions. We explored including a contact tracing sample or household study data with simulations and in the analysis of real outbreak data.

The South African H1N1 outbreak is an example of a large outbreak in which the epidemic curve contains many cases spread over time. We explored including the contact tracing sample with and without weighting the observed SIs in the prior. Using the contact tracing sample as is, which was equivalent to 0.8%-1.5% of the total outbreak size, led to similar results for the posterior estimates. When we increased the weight of the contact tracing sample to be more influential (27% of the total outbreak size), the estimates were impacted. The increase in prior information led to a slight increase in the estimates of the reproductive number and the mean of the serial interval (results shown in Table S13 in S3 Appendix). The posterior mean of μ was almost identical to the prior mean, which indicates that a heavily weighted prior can bias the posterior results. For the SARS outbreak in Singapore and Hong Kong the contact tracing sample size was much larger relative to the number of cases in epidemic curve, which also impacted our results. Here, when we informed the SI prior distribution with contact tracing data, the posterior estimates of R_0_ and μ decreased, and the posterior estimate of μ was similar to the prior mean (mean of the contact tracing sample). This decrease in the estimates is likely related to the timing of the contact tracing sample collection and of the implementation of control measures, and is attributable to including a large contact tracing sample from a different phase of the outbreak. The results from the outbreak analyses support the work of Kenah et al. [32], in which they show that when the prevalence of an infection is lower, the serial interval should be larger.

Contact tracing samples with a large number of observed SIs relative to the total outbreak size should be used with caution, especially if obtained from a different part of the outbreak. If the additional data source is substantially different from the true underlying process, the posterior estimates may be biased towards the contact tracing sample as seen with the SARS outbreak analysis, the South Africa sensitivity analysis, and prior 5 from our simulation study. We recommend analyzing the data with and without the contact tracing sample to see if the final estimates differ substantially and not using a SI prior that is weighted to be more than 4% of the total outbreak size. In instances when there are substantial differences, we recommend critical examination of the techniques used to obtain the samples and consideration of the total outbreak size and length in order to evaluate potential explanations for the inconsistencies. Ideally, one would be able to determine if the data are in conflict, or if the differences are due to an improvement in estimation because more information is being incorporated.

The SARS outbreaks highlighted the improvements of a Bayesian approach as compared to a frequentist approach. For these outbreaks, we observe more reasonable estimates for the posterior means compared to the MLEs, as well as narrower credible intervals. The White and Pagano method [6] has been shown to overestimate R_0_ as R_0_ increases [33], which is likely due to a flattening of the likelihood and the difficulty of finding a maximum using a numerical optimizer. If additional data (e.g. contact tracing) is not available for a given outbreak, in some instances, especially with a large number of parameters, a Bayesian approach with a uniform (Bayes-Laplace) prior for the SI shows improvement over the traditional White and Pagano approach.

When contact tracing data is not available or chosen to not be included in the analysis, then the Bayes-Laplace prior for the SI is recommended, which assigns ones to the hyperparameters of the Dirichlet distribution. One challenge, when there is little or no contact tracing data to inform the prior distribution of the serial interval, is to determine an appropriate value for k, the maximum SI length. We demonstrated through simulation and in the analysis of H1N1 in South Africa and SARS in Hong Kong, that the DIC can be utilized to select the best model and value of k. We recommend that a set of plausible values of k be selected, and then model comparison be done with the DIC; smaller values of DIC indicate better fit.

Our simulations also highlighted how posterior estimates can be affected by the number of observed generations of transmission. When outbreak sizes were small and reproductive numbers were large we observed short epidemic lengths, such as when N = 50 and R_0_ = 6 we only observe epidemic lengths of four to five days for an SI with maximum length five and saw that ${\hat{R}}_{0}$ is underestimated. For simulations based on larger values of the reproductive number the results were varied; the best results were observed for large epidemic sizes. Outbreaks based on smaller reproductive numbers resulted in better posterior estimates regardless of outbreak size. Griffin et al. [33] notes that estimation is better when cases are spread over many generations, as opposed to having more individuals in fewer generations, as is likely to occur with a large reproductive number. We also observed that not only is the total number of observed cases important, but also when these cases appear [13]. The issues with estimating the parameters with the larger R_0_ could be resolved by allowing the epidemic curve to cover more generations of data (larger N). In fact, one is unlikely to begin such an analysis for outbreak that has only been occurring over so few days. Additional information about the reproductive number that could be used to inform its prior distribution could potentially improve this issue as well. However, in practice, these scenarios where the SI is relatively short and the reproductive number is large are uncommon.

Realistically, the methods we have proposed are much more applicable to a setting where the reproductive number is relatively small. We are able to show when we have sufficient data from the epidemic curve and an accurate contact tracing sample that our approach is preferable to using only epidemic curve data, as demonstrated through the simulations. Our findings support those seen in Becker et al. [13], in which they conclude that having only 10 additional observations on the SI can substantially improve estimation. We also showed through simulation that our method is comparable to the Becker approach. When no contact tracing data is available the methods are mathematically equivalent, and when we incorporate observations on the SI the two approaches are very similar. The primary difference between the two methods is in how the contact tracing data is incorporated in the estimation process, and the method we propose provides a more general framework in allowing outside data sources and different prior weights to be utilized.

Although our approach has been shown to be advantageous compared to previous approaches, there are some limitations and potential improvements that could be made. First, we assume a stationary distribution for the SI, which may not be appropriate in some settings. As previously discussed, changes in the prevalence of disease can affect transmission, which then affects the SI and R_0_ estimates. One potential solution would be to modify the model to obtain time-updated estimates of the SI. This could be done in phases as prevalence of the disease changes. In addition, one could consider using a hazard-based estimator, as suggested in Kenah et al., or other likelihood based approach [32, 34]. Because our focus is in the initial exponential growth phase of the outbreak, clustering of cases and SI contraction may not have a substantial impact on estimates.

Another important limitation of our method is the use of a discrete SI distribution. In assuming a multinomial distribution for the SI, the time of infection is considered the date of report, although the actual symptom onset could have occurred any time between the prior day and the current date of report. te Beest et al. [35], introduced an interval-censored approach that considers the time of symptom onset on the interval between consecutive days. We do not account for interval censoring in our analysis, which could lead to potential biases, particularly when the serial interval is short. An interesting extension of this work would be to use a continuous SI distribution, such as the gamma distribution shown in White and Pagano [6] or other smoothness assumption and estimate the serial interval using the approach in te Beest et al. [35]. The prior distributions for the parameters of the gamma distributed serial interval could then be informed from the SI estimate corrected for interval censoring. One could potentially further modify the likelihood function or overall model formulation to allow for interval-censoring to be directly incorporated in our estimation.

The Bayesian methods presented here offer a simple solution to improving estimation of R_0_ and the serial interval. By including contact tracing data via the prior distribution for the SI, we obtain better estimates for these measures when incidence data are sparse and comparable estimates with larger epidemic sizes as compared to the traditional frequentist approach. Our approach also allows for more flexibility when contact tracing samples contain a large number of observed SIs.

## Supporting Information

S1 Appendix Simulations Description. (DOCX)

S2 Appendix Simulations Results Tables. (DOC)

S3 Appendix South Africa Sensitivity Analysis Description and Results. (DOCX)

S4 Appendix Bugs and R Code. (DOCX)

S1 FigSimulation results for ${\hat{R}}_{0}$.(DOC)

S2 FigSimulation results for $\hat{\mu }$.(DOC)
