# Supplementary material for: The Impact of Prior Information on Estimates of Disease Transmissibility Using Bayesian Tools
Source: PLoS One. 2015 Mar 20;10(3):e0118762. doi: 10.1371/journal.pone.0118762 (PMC4368801; doi:10.1371/journal.pone.0118762)
Supplement: S1 Appendix — (DOCX) [file pone.0118762.s001.docx]

**Simulation Study**

**Simulation Results**

The results from the simulations are displayed in S1 and S2 Figs. and Tables S1-S6 in Supplement 2. Figures S1 and S2 plot the posterior means for R_0_ and μ, respectively. The ranges of the posterior means across the MCMC simulations are displayed with error bars. The White and Pagano maximum likelihood estimates are included for comparison. The means and ranges of the posterior means for R_0_ and μ from the 300 MCMC simulations are displayed in Tables S1-S3. Tables S4-S6 summarize the MSE for R_0_ and μ, and the Kullback-Leibler divergence for p across simulations. Both tables include White and Pagano estimates for comparison.

See manuscript for discussion of simulation results.

**Becker Results**

The results from the Becker analysis are comparable to those from our simulations and are shown in Tables S7-S12. For all epidemic sizes and R_0_ values, the Becker results are identical to our simulation results when we use a uniform prior. This is expected with the uniform prior because they are mathematically equivalent. For priors 2-4, the Becker method has slightly larger estimates than our approach, and for prior 5, the estimates from our proposed method are slightly larger. Overall the results are consistent between approaches.
