# Supplementary material for: The Impact of Prior Information on Estimates of Disease Transmissibility Using Bayesian Tools
Source: PLoS One. 2015 Mar 20;10(3):e0118762. doi: 10.1371/journal.pone.0118762 (PMC4368801; doi:10.1371/journal.pone.0118762)
Supplement: S2 Appendix — (DOC) [file pone.0118762.s002.doc]

| **Table S1. Simulations results for N=500 using five different priors and White and Pagano**  **method.** | | | | | | | | | | | | |
| --- | --- | --- | --- | --- | --- | --- | --- | --- | --- | --- | --- | --- |
|  |  | Prior 1 |  | Prior 2 |  | Prior 3 |  | Prior 4 |  | Prior 5 |  | W&P |
| R_0_ =1.25 | $\hat{R}_{0}$ | 1.26  1.11, 1.48 |  | 1.24  1.11, 1.42 |  | 1.25  1.11, 1.44 |  | 1.25  1.11, 1.43 |  | 1.33  1.14, 1.61 |  | 1.26  1.10, 1.58 |
|  | 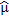 | 2.85  2.21, 3.46 |  | 2.70  2.38, 3.04 |  | 2.77  2.32, 3.25 |  | 2.79  2.42, 3.10 |  | 3.50  2.93, 3.79 |  | 2.88  1.56, 3.91 |
| R_0_ = 3 | $\hat{R}_{0}$ | 3.11  2.20, 4.21 |  | 2.93  2.47, 3.42 |  | 3.04  2.36, 3.84 |  | 3.01  2.57, 3.57 |  | 4.22  3.06, 5.27 |  | 3.54  2.00, 6.08 |
|  | 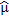 | 2.91  2.17, 3.60 |  | 2.69  2.43, 3.00 |  | 2.77  2.31, 3.34 |  | 2.78  2.50, 3.16 |  | 3.69  3.21, 3.95 |  | 3.10  1.89, 4.29 |
| R_0_ =6 | $\hat{R}_{0}$ | 5.90  1.01, 11.57 |  | 5.68  4.49, 7.03 |  | 6.00  4.29, 8.84 |  | 6.00  4.74, 7.17 |  | 9.89  5.65, 15.30 |  | 9.17  2.29, 31.37 |
|  | 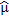 | 2.84  1.04, 3.70 |  | 2.68  2.41, 2.89 |  | 2.76  2.31, 3.23 |  | 2.77  2.46, 3.00 |  | 3.73  3.18, 4.10 |  | 3.24  1.11, 4.39 |

R_0_ varies across scenarios, as shown. True values μ and **p**: μ=2.74 and

**p**=(0.1687, 0.2102, 0.4437, 0.0642, 0.1132). Means and ranges are shown for R_0_ and μ.

| **Table S2. Simulations results for N=200 using five different priors and White and Pagano**  **Method.** | | | | | | | | | | | | |
| --- | --- | --- | --- | --- | --- | --- | --- | --- | --- | --- | --- | --- |
|  |  | Prior 1 |  | Prior 2 |  | Prior 3 |  | Prior 4 |  | Prior 5 |  | W&P |
| R_0_ =1.25 | $\hat{R}_{0}$ | 1.25  1.03, 1.52 |  | 1.24  1.03, 1.48 |  | 1.25  1.03, 1.50 |  | 1.25  1.03, 1.50 |  | 1.33  1.04, 1.69 |  | 1.27  1.03, 1.62 |
|  | 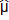 | 2.85  2.29, 3.54 |  | 2.70  2.47, 3.14 |  | 2.77  2.42, 3.36 |  | 2.78  2.52, 3.14 |  | 3.55  3.16, 3.82 |  | 2.92  1.76, 3.91 |
| R_0_ = 3 | $\hat{R}_{0}$ | 3.09  2.31, 4.05 |  | 2.90  2.41, 3.60 |  | 3.02  2.41, 3.77 |  | 2.99  2.46, 3.68 |  | 4.22  3.07, 5.79 |  | 3.69  1.62, 7.58 |
|  | 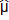 | 2.92  2.37, 3.53 |  | 2.70  2.51, 2.86 |  | 2.79  2.46, 3.05 |  | 2.79  2.55, 3.02 |  | 3.72  3.27, 3.95 |  | 3.18  1.17, 4.23 |
| R_0_ =6 | $\hat{R}_{0}$ | 6.01  3.90, 10.34 |  | 5.56  4.33, 7.26 |  | 5.90  4.25, 8.73 |  | 5.88  4.59, 7.30 |  | 9.81  5.84, 15.56 |  | 10.10  2.29, 30.86 |
|  | 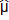 | 2.92  2.31, 3.66 |  | 2.68  2.45, 2.81 |  | 2.76  2.39, 3.02 |  | 2.78  2.55, 3.06 |  | 3.76  3.27, 4.04 |  | 3.38  1.21, 4.69 |

R_0_ varies across scenarios, as shown. True values μ and **p**: μ=2.74 and

**p**=(0.1687, 0.2102, 0.4437, 0.0642, 0.1132). Means and ranges are shown for R_0_ and μ

| **Table S3. Simulations results for N=50 using five different priors and White and Pagano**  **method.** | | | | | | | | | | | | |
| --- | --- | --- | --- | --- | --- | --- | --- | --- | --- | --- | --- | --- |
|  |  | Prior 1 |  | Prior 2 |  | Prior 3 |  | Prior 4 |  | Prior 5 |  | W&P |
| R_0_ =1.25 | $\hat{R}_{0}$ | 1.28  1.01, 2.06 |  | 1.26  1.00, 1.89 |  | 1.27  1.00, 1.96 |  | 1.27  1.01, 1.96 |  | 1.40  1.02, 2.67 |  | 1.33  1.00, 2.38 |
|  | 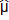 | 2.86  2.40, 3.52 |  | 2.70  2.52, 2.96 |  | 2.77  2.48, 3.18 |  | 2.77  2.60, 3.08 |  | 3.64  3.30, 3.92 |  | 3.01  1.65, 4.14 |
| R_0_ = 3 | $\hat{R}_{0}$ | 2.85  1.97, 4.48 |  | 2.72  1.95, 3.89 |  | 2.81  1.99, 4.08 |  | 2.78  1.97, 3.99 |  | 3.92  2.48, 6.33 |  | 3.79  1.68, 8.89 |
|  | 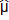 | 2.89  2.20, 3.51 |  | 2.69  2.45, 2.84 |  | 2.77  2.36, 3.02 |  | 2.77  2.53, 3.03 |  | 3.75  3.23, 3.98 |  | 3.18  1.55, 4.32 |
| R_0_ =6 | $\hat{R}_{0}$ | 5.96  3.54, 9.98 |  | 5.01  3.80, 7.31 |  | 5.45  3.77, 8.29 |  | 5.35  3.73, 7.55 |  | 9.16  5.35, 19.39 |  | 9.17  3.62, 30.11 |
|  | 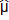 | 3.16  2.48, 3.98 |  | 2.72  2.55, 2.86 |  | 2.87  2.52, 3.20 |  | 2.83  2.55, 3.07 |  | 3.94  3.45, 4.17 |  | 3.25  2.20, 4.50 |

R_0_ varies across scenarios, as shown. True values μ and **p**: μ=2.74 and

**p**=(0.1687, 0.2102, 0.4437, 0.0642, 0.1132). Means and ranges are shown for R_0_ and μ.

| **Table S4. Simulations results continued for N=500 using five different priors and White and Pagano method.** | | | | | | | | | | | | |
| --- | --- | --- | --- | --- | --- | --- | --- | --- | --- | --- | --- | --- |
|  |  | Prior 1 |  | Prior 2 |  | Prior 3 |  | Prior 4 |  | Prior 5 |  | W&P |
| R_0_ =1.25 | MSE($\hat{R}_{0}$) | 0.004  0.000, 0.005 |  | 0.003  0.000, 0.004 |  | 0.003  0.000, 0.004 |  | 0.003  0.000, 0.004 |  | 0.012  0.001, 0.015 |  | 0.006  0.001, 0.007 |
|  | MSE(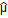) | 0.050  0.006, 0.060 |  | 0.011  0.001, 0.016 |  | 0.022  0.002, 0.026 |  | 0.012  0.001, 0.014 |  | 0.588  0.456, 0.711 |  | 0.170  0.021, 0.205 |
|  | KL div | 0.112  0.004, 0.435 |  | 0.021  0.001, 0.069 |  | 0.040  0.001, 0.145 |  | 0.094  0.010, 0.258 |  | 0.285  0.092, 0.483 |  | 0.634  0.006, 3.715 |
| R_0_ = 3 | MSE($\hat{R}_{0}$) | 0.123  0.013, 0.136 |  | 0.032  0.004, 0.047 |  | 0.051  0.004, 0.061 |  | 0.031  0.003, 0.038 |  | 1.631  0.896, 2.114 |  | 0.872  0.079, 0.896 |
|  | MSE(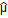) | 0.071  0.011, 0.088 |  | 0.008  0.001, 0.010 |  | 0.016  0.002, 0.016 |  | 0.008  0.001, 0.008 |  | 0.904  0.772, 1.072 |  | 0.365  0.055, 0.597 |
|  | KL div | 0.186  0.023, 0.517 |  | 0.014  0.000, 0.063 |  | 0.034  0.001, 0.189 |  | 0.149  0.021, 0.319 |  | 0.416  0.199, 0.593 |  | 0.916  0.036, 4.643 |
| R_0_ =6 | MSE($\hat{R}_{0}$) | 1.564  0.082, 1.222 |  | 0.273  0.042, 0.343 |  | 0.400  0.030, 0.440 |  | 0.162  0.011, 0.172 |  | 17.35  10.22, 21.28 |  | 24.410  1.048, 31.357 |
|  | MSE(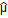) | 0.135  0.010, 0.094 |  | 0.009  0.001, 0.011 |  | 0.014  0.001, 0.017 |  | 0.007  0.001, 0.008 |  | 0.987  0.858, 1.141 |  | 0.632  0.062, 1.153 |
|  | KL div | 0.294  0.013, 3.113 |  | 0.014  0.000, 0.055 |  | 0.037  0.003, 0.139 |  | 0.171  0.039, 0.268 |  | 0.461  0.191, 0.711 |  | 1.063  0.076, 4.576 |

R_0_ varies across scenarios, as shown. True values μ and **p**: μ=2.74 and **p**=(0.1687, 0.2102, 0.4437, 0.0642, 0.1132).

Mean and IQR of the MSE for R_0_ and μ, and Kullback-Leibler divergence means and ranges for **p** are shown.

| **Table S5. Simulations results continued for N=200 using five different priors and White and Pagano method.** | | | | | | | | | | | | |
| --- | --- | --- | --- | --- | --- | --- | --- | --- | --- | --- | --- | --- |
|  |  | Prior 1 |  | Prior 2 |  | Prior 3 |  | Prior 4 |  | Prior 5 |  | W&P |
| R_0_ =1.25 | MSE($\hat{R}_{0}$) | 0.009  0.001, 0.010 |  | 0.007  0.001, 0.010 |  | 0.008  0.001, 0.011 |  | 0.008  0.001, 0.011 |  | 0.022  0.001, 0.027 |  | 0.012  0.001, 0.016 |
|  | MSE(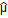) | 0.051  0.005, 0.065 |  | 0.010  0.001, 0.012 |  | 0.019  0.002, 0.023 |  | 0.010  0.001, 0.013 |  | 0.660  0.520, 0.798 |  | 0.207  0.025, 0.280 |
|  | KL div | 0.127  0.010, 0.515 |  | 0.019  0.001, 0.089 |  | 0.039  0.002, 0.180 |  | 0.107  0.015, 0.278 |  | 0.316  0.139, 0.564 |  | 0.747  0.013, 3.715 |
| R_0_ = 3 | MSE($\hat{R}_{0}$) | 0.125  0.011, 0.146 |  | 0.058  0.008, 0.080 |  | 0.068  0.008, 0.088 |  | 0.054  0.006, 0.073 |  | 1.721  0.736, 2.236 |  | 1.203  0.073, 1.206 |
|  | MSE(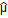) | 0.068  0.013, 0.098 |  | 0.006  0.001, 0.007 |  | 0.013  0.001, 0.018 |  | 0.008  0.001, 0.011 |  | 0.962  0.842, 1.089 |  | 0.449  0.075, 0.681 |
|  | KL div | 0.192  0.017, 0.595 |  | 0.015  0.001, 0.086 |  | 0.035  0.002, 0.200 |  | 0.154  0.040, 0.269 |  | 0.430  0.221, 0.649 |  | 1.285  0.052, 4.716 |
| R_0_ =6 | MSE($\hat{R}_{0}$) | 11.72  1.405, 23.83 |  | 11.56  2.84, 23.94 |  | 11.59  2.279, 23.83 |  | 11.82  1.95, 23.95 |  | 10.11  0.87, 23.88 |  | 40.022  2.541, 48.784 |
|  | MSE(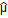) | 1.339  0.111, 2.846 |  | 1.332  0.144, 2.765 |  | 1.339  0.126, 2.849 |  | 1.393  0.147, 2.829 |  | 1.084  0.053, 2.779 |  | 0.943  0.214, 1.480 |
|  | KL div | 1.606  0.040, 5.597 |  | 1.295  0.010, 4.828 |  | 1.46  0.035, 5.283 |  | 1.497  0.029, 5.575 |  | 1.57  0.065, 6.032 |  | 1.474  0.017, 5.459 |

R_0_ varies across scenarios, as shown. True values μ and **p**: μ=2.74 and **p**=(0.1687, 0.2102, 0.4437, 0.0642, 0.1132).

Mean and IQR of the MSE for R_0_ and μ, and Kullback-Leibler divergence means and ranges for **p** are shown.

| **Table S6. Simulations results continued for N=50 using five different priors and White and Pagano method.** | | | | | | | | | | | | |
| --- | --- | --- | --- | --- | --- | --- | --- | --- | --- | --- | --- | --- |
|  |  | Prior 1 |  | Prior 2 |  | Prior 3 |  | Prior 4 |  | Prior 5 |  | W&P |
| R_0_ =1.25 | MSE($\hat{R}_{0}$) | 0.029  0.002, 0.029 |  | 0.023  0.002, 0.026 |  | 0.026  0.002, 0.028 |  | 0.026  0.002, 0.028 |  | 0.080  0.004, 0.073 |  | 0.055  0.003, 0.041 |
|  | MSE(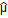) | 0.049  0.005, 0.063 |  | 0.007  0.001, 0.010 |  | 0.014  0.002, 0.015 |  | 0.006  0.001, 0.008 |  | 0.813  0.666, 0.945 |  | 0.314  0.040, 0.453 |
|  | KL div | 0.164  0.007, 0.583 |  | 0.016  0.001, 0.073 |  | 0.034  0.001, 0.121 |  | 0.140  0.033, 0.279 |  | 0.383  0.198, 0.582 |  | 1.181  0.022, 5.020 |
| R_0_ = 3 | MSE($\hat{R}_{0}$) | 0.247  0.029, 0.340 |  | 0.215  0.035, 0.339 |  | 0.203  0.025, 0.318 |  | 0.206  0.024, 0.321 |  | 1.392  0.137, 1.954 |  | 2.526  0.128, 1.857 |
|  | MSE(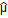) | 0.058  0.007, 0.081 |  | 0.006  0.001, 0.008 |  | 0.011  0.001, 0.014 |  | 0.006  0.000, 0.005 |  | 1.017  0.926, 1.132 |  | 0.555  0.072, 0.913 |
|  | KL div | 0.224  (0.026, 0.614 |  | 0.012  0.001, 0.090 |  | 0.030  0.001, 0.196 |  | 0.181  0.028, 0.300 |  | 0.463  0.246, 0.636 |  | 1.511  0.021, 5.512 |
| R_0_ =6 | MSE($\hat{R}_{0}$) | 1.360  0.151, 1.654 |  | 1.312  0.463, 1.885 |  | 0.859  0.141, 1.329 |  | 0.939  0.135, 1.346 |  | 13.70  3.511, 16.40 |  | 26.616  0.506, 23.345 |
|  | MSE(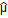) | 0.215  0.077, 0.253 |  | 0.002  0.000, 0.002 |  | 0.023  0.006, 0.027 |  | 0.012  0.003, 0.013 |  | 1.432  1.338, 1.533 |  | 0.494  0.040, 0.701 |
|  | KL div | 0.327  0.051, 0.955 |  | 0.011  0.001, 0.049 |  | 0.045  0.004, 0.198 |  | 0.198  0.082, 0.290 |  | 0.590  0.318, 0.865 |  | 1.109  0.037, 5.578 |

R_0_ varies across scenarios, as shown. True values μ and **p**: μ=2.74 and **p**=(0.1687, 0.2102, 0.4437, 0.0642, 0.1132).

Mean and IQR of the MSE for R_0_ and μ, and Kullback-Leibler divergence means and ranges for **p** are shown.

| **Table S7. Simulations results for N=500 using five different priors using Becker et al.**  **method.** | | | | | | | | | | |
| --- | --- | --- | --- | --- | --- | --- | --- | --- | --- | --- |
|  |  | Prior 1 |  | Prior 2 |  | Prior 3 |  | Prior 4 |  | Prior 5 |
| R_0_ =1.25 | $\hat{R}_{0}$ | 1.26  1.11, 1.48 |  | 1.25  1.11, 1.43 |  | 1.26  1.11, 1.46 |  | 1.25  1.12, 1.44 |  | 1.32  1.14, 1.59 |
|  | 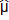 | 2.85  2.21, 3.46 |  | 2.77  2.46, 3.03 |  | 2.84  2.42, 3.20 |  | 2.82  2.48, 3.08 |  | 3.44  3.05, 3.70 |
| R_0_ = 3 | $\hat{R}_{0}$ | 3.11  2.20, 4.21 |  | 2.99  2.54, 3.47 |  | 3.11  2.48, 3.68 |  | 3.04  2.61, 3.50 |  | 4.04  3.19, 4.94 |
|  | 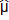 | 2.91  2.17, 3.60 |  | 2.77  2.53, 3.00 |  | 2.87  2.48, 3.25 |  | 2.82  2.57, 3.13 |  | 3.60  3.21, 3.83 |
| R_0_ =6 | $\hat{R}_{0}$ | 5.90  1.01, 11.57 |  | 5.83  4.64, 7.13 |  | 6.20  4.53, 8.47 |  | 6.06  4.85, 7.19 |  | 9.17  5.70, 13.32 |
|  | 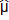 | 2.84  1.04, 3.70 |  | 2.75  2.50, 2.94 |  | 2.86  2.46, 3.20 |  | 2.82  2.53, 3.02 |  | 3.63  3.15, 3.95 |

R_0_ varies across scenarios, as shown. True values μ and **p**: μ=2.74 and

**p**=(0.1687, 0.2102, 0.4437, 0.0642, 0.1132). Means and ranges are shown for R_0_ and μ

| **Table S8. Simulations results for N=200 using five different priors using Becker et al.**  **method.** | | | | | | | | | | |
| --- | --- | --- | --- | --- | --- | --- | --- | --- | --- | --- |
|  |  | Prior 1 |  | Prior 2 |  | Prior 3 |  | Prior 4 |  | Prior 5 |
| R_0_ =1.25 | $\hat{R}_{0}$ | 1.25  1.03, 1.52 |  | 1.24  1.03, 1.50 |  | 1.25  1.03, 1.52 |  | 1.25  1.12, 1.44 |  | 1.32  1.04, 1.68 |
|  | 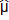 | 2.85  2.29, 3.54 |  | 2.76  2.55, 3.14 |  | 2.85  2.54, 3.31 |  | 2.82  2.48, 3.08 |  | 3.48  3.15, 3.73 |
| R_0_ = 3 | $\hat{R}_{0}$ | 3.09  2.31, 4.05 |  | 2.96  2.45, 3.62 |  | 3.08  2.47, 3.80 |  | 3.01  2.50, 3.71 |  | 4.02  3.00, 5.39 |
|  | 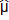 | 2.92  2.37, 3.53 |  | 2.77  2.58, 2.92 |  | 2.88  2.58, 3.08 |  | 2.83  2.62, 3.02 |  | 3.62  3.24, 3.82 |
| R_0_ =6 | $\hat{R}_{0}$ | 6.01  3.90, 10.34 |  | 5.67  4.52, 7.29 |  | 6.07  4.54, 8.53 |  | 5.93  4.64, 7.34 |  | 9.02  5.77, 13.58 |
|  | 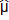 | 2.92  2.31, 3.66 |  | 2.76  2.56, 2.87 |  | 2.86  2.55, 3.06 |  | 2.82  2.62, 3.05 |  | 3.66  3.15, 3.91 |

R_0_ varies across scenarios, as shown. True values μ and **p**: μ=2.74 and

**p**=(0.1687, 0.2102, 0.4437, 0.0642, 0.1132). Means and ranges are shown for R_0_ and μ

| **Table S9.** **Simulations results for N=50 using five different priors using Becker et al.**  **method.** | | | | | | | | | | |
| --- | --- | --- | --- | --- | --- | --- | --- | --- | --- | --- |
|  |  | Prior 1 |  | Prior 2 |  | Prior 3 |  | Prior 4 |  | Prior 5 |
| R_0_ =1.25 | $\hat{R}_{0}$ | 1.28  1.01, 2.061 |  | 1.27  1.01, 1.92 |  | 1.28  1.00, 2.00 |  | 1.28  1.00, 1.98 |  | 1.38  1.02, 2.56 |
|  | 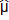 | 2.86  2.40, 3.52 |  | 2.76  2.61, 2.98 |  | 2.85  2.63, 3.15 |  | 2.81  2.65, 3.07 |  | 3.55  3.27, 3.80 |
| R_0_ = 3 | $\hat{R}_{0}$ | 2.85  1.97, 4.48 |  | 2.76  1.99, 3.95 |  | 2.87  2.04, 4.14 |  | 2.81  1.99, 4.05 |  | 3.73  2.41, 5.80 |
|  | 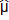 | 2.89  2.20, 3.51 |  | 2.76  2.54, 2.90 |  | 2.86  2.51, 3.04 |  | 2.82  2.60, 3.00 |  | 3.64  3.21, 3.83 |
| R_0_ =6 | $\hat{R}_{0}$ | 5.96  3.54, 9.98 |  | 5.10  3.84, 7.50 |  | 5.51  3.97, 8.33 |  | 5.35  3.79, 7.69 |  | 8.23  5.20, 15.49 |
|  | 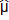 | 3.16  2.48, 3.98 |  | 2.80  2.63, 2.93 |  | 2.95  2.64, 3.19 |  | 2.87  2.64, 3.07 |  | 3.80  3.39, 4.01 |

R_0_ varies across scenarios, as shown. True values μ and **p**: μ=2.74 and

**p**=(0.1687, 0.2102, 0.4437, 0.0642, 0.1132). Means and ranges are shown for R_0_ and μ

| **Table S10. Simulations results continued for N=500 using five different priors using Becker et al. method.** | | | | | | | | | | |
| --- | --- | --- | --- | --- | --- | --- | --- | --- | --- | --- |
|  |  | Prior 1 |  | Prior 2 |  | Prior 3 |  | Prior 4 |  | Prior 5 |
| R_0_ =1.25 | MSE($\hat{R}_{0}$) | 0.004  0.000, 0.005 |  | 0.003  0.000, 0.004 |  | 0.004  0.000, 0.004 |  | 0.003  0.000, 0.004 |  | 0.011  0.001, 0.014 |
|  | MSE(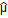) | 0.050  0.006, 0.060 |  | 0.001  0.001, 0.008 |  | 0.024  0.003, 0.031 |  | 0.013  0.002, 0.018 |  | 0.495  0.387, 0.593 |
|  | KL div | 0.112  0.004, 0.435 |  | 0.017  0.000, 0.069 |  | 0.041  0.003, 0.153 |  | 0.098  0.014, 0.234 |  | 0.258  0.082, 0.436 |
| R_0_ = 3 | MSE($\hat{R}_{0}$) | 0.123  0.013, 0.136 |  | 0.027  0.003, 0.035 |  | 0.056  0.006, 0.071 |  | 0.030  0.003, 0.037 |  | 1.179  0.650, 1.529 |
|  | MSE(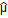) | 0.071  0.011, 0.088 |  | 0.005  0.001, 0.005 |  | 0.026  0.006, 0.034 |  | 0.011  0.002, 0.014 |  | 0.739  0.635, 0.869 |
|  | KL div | 0.186  0.023, 0.517 |  | 0.012  0.001, 0.064 |  | 0.045  0.008, 0.171 |  | 0.149  0.022, 0.287 |  | 0.366  0.188, 0.513 |
| R_0_ =6 | MSE($\hat{R}_{0}$) | 1.564  0.082, 1.222 |  | 0.186  0.014, 0.236 |  | 0.369  0.030, 0.444 |  | 0.150  0.011, 0.178 |  | 11.37  6.943, 14.17 |
|  | MSE(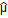) | 0.135  0.010, 0.094 |  | 0.004  0.000, 0.004 |  | 0.024  0.007, 0.033 |  | 0.010  0.003, 0.014 |  | 0.806  0.712, 0.927 |
|  | KL div | 0.294  0.013, 3.113 |  | 0.014  0.001, 0.058 |  | 0.053  0.011, 0.157 |  | 0.170  0.042, 0.248 |  | 0.406  0.182, 0.597 |

R_0_ varies across scenarios, as shown. True values μ and **p**: μ=2.74 and **p**=(0.1687, 0.2102, 0.4437, 0.0642, 0.1132).

Mean and IQR of the MSE for R_0_ and μ, and Kullback-Leibler divergence means and ranges for **p** are shown.

| **Table S11. Simulations results continued for N=200 using five different priors using Becker et al. method.** | | | | | | | | | | |
| --- | --- | --- | --- | --- | --- | --- | --- | --- | --- | --- |
|  |  | Prior 1 |  | Prior 2 |  | Prior 3 |  | Prior 4 |  | Prior 5 |
| R_0_ =1.25 | MSE($\hat{R}_{0}$) | 0.009  0.001, 0.010 |  | 0.007  0.001, 0.010 |  | 0.008  0.001, 0.010 |  | 0.003  0.000, 0.004 |  | 0.020  0.001, 0.024 |
|  | MSE(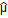) | 0.051  0.005, 0.065 |  | 0.007  0.001, 0.008 |  | 0.023  0.003, 0.032 |  | 0.013  0.002, 0.018 |  | 0.548  0.440, 0.653 |
|  | KL div | 0.127  0.010, 0.515 |  | 0.016  0.001, 0.087 |  | 0.042  0.006, 0.172 |  | 0.098  0.014, 0.234 |  | 0.284  0.123, 0.487 |
| R_0_ = 3 | MSE($\hat{R}_{0}$) | 0.125  0.011, 0.146 |  | 0.052  0.006, 0.070 |  | 0.074  0.006, 0.082 |  | 0.053  0.005, 0.066 |  | 1.222  0.500, 1.611 |
|  | MSE(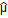) | 0.068  0.013, 0.098 |  | 0.004  0.000, 0.006 |  | 0.026  0.009, 0.036 |  | 0.012  0.003, 0.016 |  | 0.778  0.691, 0.876 |
|  | KL div | 0.192  0.017, 0.595 |  | 0.013  0.001, 0.074 |  | 0.046  0.008, 0.155 |  | 0.153  0.047, 0.249 |  | 0.377  0.213, 0.614 |
| R_0_ =6 | MSE($\hat{R}_{0}$) | 1.163  0.126, 1.587 |  | 0.359  0.052, 0.503 |  | 0.506  0.038, 0.759 |  | 0.263  0.023, 0.364 |  | 11.10  5.206, 14.06 |
|  | MSE(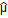) | 0.074  0.011, 0.102 |  | 0.004  0.000, 0.005 |  | 0.024  0.007, 0.036 |  | 0.011  0.003, 0.014 |  | 0.846  0.740, 0.986 |
|  | KL div | 0.255  0.053, 0.638 |  | 0.015  0.001, 0.081 |  | 0.055  0.017, 0.171 |  | 0.181  0.052, 0.279 |  | 0.422  0.266, 0.601 |

R_0_ varies across scenarios, as shown. True values μ and **p**: μ=2.74 and **p**=(0.1687, 0.2102, 0.4437, 0.0642, 0.1132).

Mean and IQR of the MSE for R_0_ and μ, and Kullback-Leibler divergence means and ranges for **p** are shown.

| **Table S12. Simulations results continued for N=50 using five different priors using Becker et al. method.** | | | | | | | | | | |
| --- | --- | --- | --- | --- | --- | --- | --- | --- | --- | --- |
|  |  | Prior 1 |  | Prior 2 |  | Prior 3 |  | Prior 4 |  | Prior 5 |
| R_0_ =1.25 | MSE($\hat{R}_{0}$) | 0.029  0.002, 0.029 |  | 0.025  0.002, 0.027 |  | 0.028  0.002, 0.030 |  | 0.027  0.002, 0.028 |  | 0.070  0.003, 0.063 |
|  | MSE(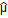) | 0.049  0.005, 0.063 |  | 0.004  0.000, 0.005 |  | 0.019  0.003, 0.028 |  | 0.009  0.001, 0.012 |  | 0.657  0.554, 0.750 |
|  | KL div | 0.164  0.007, 0.583 |  | 0.013  0.001, 0.055 |  | 0.041  0.007, 0.119 |  | 0.140  0.033, 0.261 |  | 0.338  0.189, 0.491 |
| R_0_ = 3 | MSE($\hat{R}_{0}$) | 0.247  0.029, 0.340 |  | 0.197  0.023, 0.309 |  | 0.189  0.020, 0.289 |  | 0.195  0.022, 0.303 |  | 0.963  0.083, 1.347 |
|  | MSE(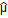) | 0.058  0.007, 0.081 |  | 0.003  0.000, 0.004 |  | 0.021  0.006, 0.030 |  | 0.009  0.002, 0.011 |  | 0.808  0.734, 0.901 |
|  | KL div | 0.224  0.026, 0.614 |  | 0.011  0.001, 0.080 |  | 0.045  0.008, 0.171 |  | 0.174  0.029, 0.268 |  | 0.402  0.199, 0.531 |
| R_0_ =6 | MSE($\hat{R}_{0}$) | 1.360  0.151, 1.654 |  | 1.148  0.377, 1.662 |  | 0.754  0.089, 1.143 |  | 0.906  0.124, 1.363 |  | 7.158  1.453, 9.038 |
|  | MSE(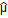) | 0.215  0.077, 0.253 |  | 0.004  0.001, 0.005 |  | 0.047  0.029, 0.054 |  | 0.019  0.010, 0.022 |  | 1.113  1.043, 1.181 |
|  | KL div | 0.327  0.051, 0.955 |  | 0.013  0.002, 0.056 |  | 0.063  0.020, 0.172 |  | 0.190  0.093, 0.264 |  | 0.491  0.286, 0.696 |

R_0_ varies across scenarios, as shown. True values μ and **p**: μ=2.74 and **p**=(0.1687, 0.2102, 0.4437, 0.0642, 0.1132).

Mean and IQR of the MSE for R_0_ and μ, and Kullback-Leibler divergence means and ranges for **p** are shown.
