# Supplementary material for: The Impact of Prior Information on Estimates of Disease Transmissibility Using Bayesian Tools
Source: PLoS One. 2015 Mar 20;10(3):e0118762. doi: 10.1371/journal.pone.0118762 (PMC4368801; doi:10.1371/journal.pone.0118762)
Supplement: S3 Appendix — (DOCX) [file pone.0118762.s003.docx]

**South Africa Influenza A(H1N1)2009pdm Sensitivity Analysis.**

The results for the influenza outbreak in South Africa were not affected by the inclusion of contact tracing data; however a difference was seen for the SARS outbreak results in Hong Kong and Singapore. This difference is likely due to the size of the contact tracing sample relative to the total epidemic size. A larger contact tracing sample provides more weight in the prior, which could affect the posterior estimates. To explore this we include an additional analysis that evaluates the influence of weighting the contact tracing data in the prior distribution of the South Africa analysis.

The contact tracing samples used in the initial analysis were based on actual observed serial intervals and corresponded to only 0.78%-1.53% of the total outbreak size, thus only had a small influence on the posterior distribution. In this sensitivity analysis we weight the contact tracing samples such that the prior sample size totals 27% of the total outbreak size, which is similar to the SARS outbreak in Hong Kong.

When we weight the prior to be 27% of the total outbreak size, the estimates of R_0_ and µ are impacted (results shown in Table S13). The estimates of µ are slightly larger and are more similar to their prior means. The means of the contact tracing samples (priors) for the confirmed ILI, probable ILI, and Australia samples are 2.31, 2.69, and 2.89, respectively. The posterior means are almost identical to these values with estimates of 2.28, 2.64, and 2.84, respectively. The estimates of R_0_ are also increased, likely due to the relatedness of the two measures.

| **Table S13.** **Means and 95% credible intervals for South Africa Influenza A(H1N1)2009pdm comparing informative prior to a heavily weighed informative prior.** | | | | | | |
| --- | --- | --- | --- | --- | --- | --- |
|  | Informative Prior with Observed SIs (weight = 1%) | | | Informative Prior with Large Weight  (Weight = 27%) | | |
|  | C ILI | P ILI | Australia | C ILI | P ILI | Australia |
| R_0_ | **1.36**  1.27, 1.45 | **1.41**  1.32, 1.51 | **1.43**  1.35, 1.53 | **1.41**  1.36, 1.47 | **1.48**  1.42, 1.54 | **1.53**  1.47, 1.60 |
| μ | **2.00**  1.62, 2.42 | **2.33**  1.92, 2.81 | **2.42**  2.06, 2.85 | **2.28**  2.18, 2.37 | **2.64**  2.53, 2.75 | **2.84**  2.73, 2.94 |
| p_1_ | 0.55  0.43, 0.67 | 0.45  0.38, 0.61 | 0.42  0.31, 0.52 | 0.39  0.35, 0.42 | 0.33  0.30, 0.37 | 0.19  0.16, 0.22 |
| p_2_ | 0.19  0.09, 0.32 | 0.15  0.07, 0.25 | 0.21  0.12, 0.31 | 0.26  0.23, 0.29 | 0.17  0.14, 0.20 | 0.27  0.24, 0.30 |
| p_3_ | 0.04  0.01, 0.11 | 0.08  0.03, 0.15 | 0.11  0.06, 0.19 | 0.10  0.08, 0.12 | 0.16  0.14, 0.19 | 0.23  0.20, 0.26 |
| p_4_ | 0.12  0.04, 0.23 | 0.14  0.06, 0.24 | 0.11  0.05, 0.20 | 0.20  0.17, 0.23 | 0.23  0.20, 0.26 | 0.18  0.15, 0.21 |
| p_5_ | 0.09  0.00, 0.22 | 0.07  0.01, 0.18 | 0.11  0.04, 0.20 | 0.05  0.04, 0.07 | 0.07  0.05, 0.09 | 0.11  0.09, 0.13 |
| p_6_ |  | 0.06  0.00, 0.18 | 0.04  0.00, 0.14 |  | 0.04  0.02, 0.05 | 0.03  0.02, 0.04 |

Australia: Australian Contact Trace data; C ILI: Confirmed Influenza-Like Illness; P ILI: Probable Influenza-Like Illness.
