# Supplementary material for: The Impact of Prior Information on Estimates of Disease Transmissibility Using Bayesian Tools
Source: PLoS One. 2015 Mar 20;10(3):e0118762. doi: 10.1371/journal.pone.0118762 (PMC4368801; doi:10.1371/journal.pone.0118762)
Supplement: S4 Appendix — (DOCX) [file pone.0118762.s004.docx]

BUGS and R code

#########################

# White and Pagano Model #

#########################

model{

for (k in 1:K) {

p[k] <- delta[k] / sum(delta[])

delta[k] ~ dgamma(alpha[k],1)

}

for (j in 1:K) {

w[j] <- j

}

mu<- inprod(w[],p[])

for (i in K:(T, 1+K)) {

Ns[i]<- N[i, K+1]

}

for (i in 1:(K, 1)) {

Ns[i]<- 0

}

for (t in 1:T) {

for (j in 1:K) {

NN[t,j]<- Ns[t, j+K]

} }

for (t in 1:T) {

muN[t+1]<- R*inprod(NN[t,],p[])

}

for (t in 2:T) {

M[t, 1] ~ dpois(muN[t])

}

log(R) <- logR

logR ~ dnorm(0,1.0E, 6)

}

#############################

# R code with BRUGS Package #

#############################

library(BRugs)

for (i in 1:300){

modelCheck("Model.txt")

modelData(“Data.txt”)

modelCompile(1)

modelInits(“Initials.txt”)

samplesSet(c('mu', 'p', 'R'))

modelUpdate(20000, thin=20)

samplesSetBeg(10000)

samplesSetEnd(20000)

sumstats11[[i]] <- samplesStats('*')

means11[[i]] <- sumstats11[[i]][,1]

R = samplesSample('R')

mu = samplesSample('mu')

p1 = samplesSample('p[1]')

p2 = samplesSample('p[2]')

p3 = samplesSample('p[3]')

p4 = samplesSample('p[4]')

p5 = samplesSample('p[5]')

post.samples = data.frame(R, mu, p1, p2, p3, p4, p5)

}
