# Supplementary material for: The Impact of Prior Information on Estimates of Disease Transmissibility Using Bayesian Tools
Source: PLoS One. 2015 Mar 20;10(3):e0118762. doi: 10.1371/journal.pone.0118762 (PMC4368801; doi:10.1371/journal.pone.0118762)
Supplement: S2 Fig — (DOC) [file pone.0118762.s006.doc]

**Fig. S2. Simulation results for
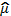
.**


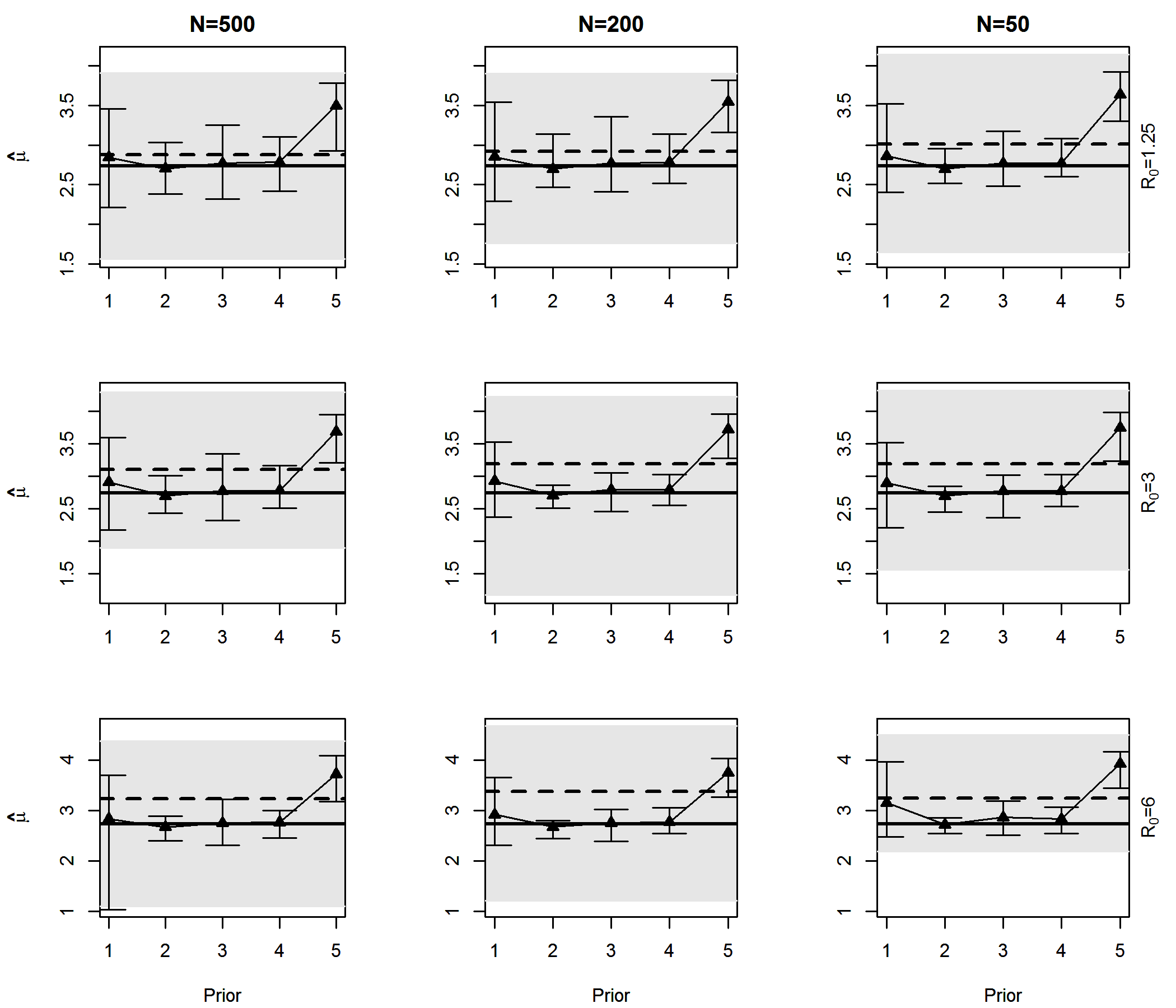


Each row shows results for a different R_0_ (row 1: R_0_ = 1.25; row 2: R_0_ = 3; row 3: R_0_ = 6). Each column shows results for a different N (column 1: N=500; column 2: N=200, column 3: N=50). The mean for each prior is plotted as a black triangle and the ranges are shown as error bars. The solid black line represents the true µ and the White and Pagano estimate is displayed with the dashed line. The gray shaded region is the range of values for the White and Pagano estimates.
